# Trends in antimicrobial susceptibility patterns in Salmonella from human and nonhuman sources in Sao Paulo State, Brazil, 2016-2023

**DOI:** 10.1590/S1678-9946202466064

**Published:** 2024-11-11

**Authors:** Monique Ribeiro Tiba-Casas, Elisabete Aparecida Almeida, Gisele Lozano Costa, Amanda Maria de Jesus Bertani, Thais Vieira, Carlos Henrique Camargo

**Affiliations:** 1 Instituto Adolfo Lutz, Centro de Bacteriologia, São Paulo, São Paulo, Brazil

**Keywords:** Salmonella, Multi-drug resistance, Antimicrobial resistance, Serotyping

## Abstract

Antimicrobial resistance constitutes a significant global challenge to public health and development, in which non-typhoidal *Salmonella* emerges as a critical concern. This study investigates the prevalence and antimicrobial resistance profiles of *Salmonella* isolates from both human and nonhuman sources. A total of 2,511 *Salmonella* isolates that had been collected from 2016 to 2023 were analyzed, of which 1,724 underwent antimicrobial susceptibility testing. The main focus lied on the 10 most prevalent serotypes, totaling 957 isolates. Serotyping showed the diverse distribution of serotypes, with Heidelberg, Typhimurium, Enteritidis, and the monophasic *Salmonella* Typhimurium occurring most often. Antimicrobial resistance was common since 512 strains resisted at least one drug and 319 several drugs. Notably, the Heidelberg and Mbandaka serotypes, predominantly occurring in nonhuman samples, showed multidrug resistance. *Salmonella* Typhi remained susceptible to antimicrobials. Resistance to nalidixic acid, tetracycline, sulfonamides, and ampicillin was prevalent, whereas all isolates remained susceptible to imipenem. A reduction in susceptibility rates for aminoglycosides was observed over the study period. Extended-spectrum β-lactamase production occurred in 4.4% of the isolates, of which Heidelberg configured the most prevalent extended-spectrum β-lactamase-positive serotype. These findings underscore the importance of surveillance and effective monitoring to control this pathogen, highlighting the necessity of prioritizing public health efforts.

## INTRODUCTION

Antimicrobial resistance is one of the most significant global challenges to public health and development worldwide. Increasing antimicrobial resistance in non-typhoidal *Salmonella* represents a critical concern for global public health^1,2^. It often constitutes a significant foodborne pathogen associated with gastrointestinal disorders, various localized infections, and bacteremia^3^. Moreover, the levels of antimicrobial resistance in *Salmonella* isolates vary across strains, serovars, geographic locations, and host sources^4^.

The indiscriminate application of antibiotics in human and animal health and food production and their subsequent leaching into the environment have contributed to increasing antimicrobial resistance bacteria. In recent years, the use of antimicrobials in production animals and the emergence of antimicrobial resistance have garnered considerable attention in public health discourse. There is growing apprehension regarding the potential transmission of resistance genes or resistant pathogens from animals to humans via the food chain^5^. The situation regarding *Salmonella* is particularly intricate as administering antibiotics for treatment or prophylaxis in veterinary medicine and their inclusion as growth promoters in animal feed may promote the emergence of resistance^1,6^, offering a potential hazard to public health due to the risk of zoonotic infections.

This study aimed to investigate the prevalence and antimicrobial resistance profiles of *Salmonella* isolates from both human and nonhuman sources. Our findings underscore the importance of surveillance and effective monitoring as essential measures to combat antimicrobial resistance in microorganisms associated with foodborne infections, emphasizing the significance of prioritizing public health efforts.

## MATERIAL AND METHODS

### Bacterial isolates

In this study, 2,511 *Salmonella* isolates that were collected from human infections and nonhuman sources from 2016 to 2023 were analyzed. These isolates were sent to our laboratory for serotyping since the Adolfo Lutz Institute is a reference laboratory in public health in Brazil. Antimicrobial susceptibility testing was conducted on 1,724 isolates encompassing all human isolates, which comprised 86 serotypes. Thus, the main focus of this study was to evaluate the results of antimicrobial resistance in the 10 most prevalent serotypes in the region, totaling 957 isolates.

Overall, 957 isolates, representing 55.5% of the total number of isolates that underwent antimicrobial susceptibility testing, were identified as one of the 10 most prevalent serotypes in our region and were further examined. The prevalence of isolates from human sources was initially attested: blood (374, 39%), stool (116, 12.1%), urine (46, 4.8%), and body fluids (39, 4%). Chicken (213, 22.3%) and three isolates from peanuts were identified from food sources. Additionally, 35 isolates from drag swabs and five from sewage sludge were received and characterized as environmental isolates (40, 4.2%). Finally, poultry (92, 9.6%) and 34 isolates (34, 3.6%) from swine, foals, and dogs as isolates from animal source were identified.

### Serotyping

The isolates were serotyped, which involved characterizing somatic O antigens and phase 1 and phase 2 flagellar H antigens by agglutination tests with specific antisera. The antisera were prepared at the Laboratory of Enteric Pathogens at Instituto Adolfo Lutz in Sao Paulo, following the guidelines outlined in the Kauffmann–White-Le Minor scheme for *Salmonella* serotyping^7^. This study focused on 957 isolates representing the 10 most commonly occurring serotypes for which antimicrobial susceptibility tests were conducted, namely: Typhimurium, Heidelberg, *S*.I.4,[5],12:i:-, Enteritidis, Mbandaka, Dublin, Infantis, Newport, Typhi, and Saintpaul.

### Antimicrobial susceptibility testing

Antimicrobial susceptibility tests were performed by the disk diffusion method according to the Clinical and Laboratory Standards Institute guidelines and interpretation criteria^8^. The following antimicrobial disks were tested: penicillin (ampicillin (10 µg)), β-lactam combination agents (amoxicillin/clavulanic acid (20/10 µg)), cephems (ceftazidime (30 µg), cefotaxime (30 µg), ceftriaxone (30 µg), cefepime (30 µg), cefoxitin (30 µg)), monobactams (aztreonam (30 µg)), carbapenems (Imipenem (10 µg)), aminoglycosides (amikacin (30 µg), gentamicin (10 µg), streptomycin (10 µg)), tetracyclines (tetracycline (30 µg)), quinolones and fluoroquinolones (nalidixic acid (30µg), (ciprofloxacin (5 µg), pefloxacin (5 µg)), folate pathway antagonists (trimethoprim/sulfamethoxazole (1.25/23.75 µg), sulfonamide (250 µg)), and phenicols (chloramphenicol (30 µg)).

Quality control results for the disk diffusion tests remained within acceptable quality control ranges according to Clinical and Laboratory Standards Institute guidelines. *Escherichia coli* ATCC 25992 and *Pseudomonas aeruginosa* ATCC 27853 were used as controls on each test.

### Extended-spectrum β-lactamase (ESBL) detection by the double-disk synergy test

ESBL production in *Salmonella* spp. was identified by the double-disk synergy test. The Mueller-Hinton agar was inoculated with a standardized inoculum of *Salmonella* (corresponding to 0.5 McFarland tube). The amoxicillin plus clavulanic acid (AMX/AC; 20/10 µg) disk was inserted in the plate center and four test disks of ceftazidime (CAZ 30 µg), ceftriaxone (CRO 30 µg), cefotaxime (CTX 30 µg), and aztreonam (ATM 30 µg) disks were placed 20 mm apart from the amoxicillin clavulanic acid disk. The plates were incubated overnight at 37°C. The enhancement of the inhibition zone of any tested disk toward the amoxicillin–clavulanic acid proposed the presence of extended-spectrum beta-lactamases^9,10^.

## RESULTS

This study investigated the antimicrobial resistance rates of various *Salmonella* serotypes from several sources the Instituto Adolfo Lutz received from 2016 to 2023. During this period, 2,511 received isolates underwent serotyping. While serotypes were identified for all 2,511 isolates, antimicrobial susceptibility testing was conducted on 1,724 of these isolates (including all human ones), representing 86 serotypes. This study only describes the antimicrobial resistance profiles for the 10 most common serotypes. The following serotypes occurred most often: Heidelberg, Typhimurium, Enteritidis, and the Monophasic *Salmonella* Typhimurium (Supplementary Table S1).

As the main objective of this study was to focus on the human isolates and the most frequent serotypes, the 935 clinical non-duplicate *Salmonella* spp. isolates primarily stemmed from various sources, including blood (569), stool (204), urine (90), and other body fluids or from unspecified origins (72). Additionally, 789 *Salmonella*spp. isolates stemmed from nonhuman sources, such as animals (291) — predominantly poultry, pigs, and cattle —, food (360) — including food-producing animals and other foodstuffs, the environment (138) — predominantly in poultry production —, and sewage or drag swab sources.

This study identified 957 isolates (corresponding to 55.5% of the total number of isolates that underwent antimicrobial susceptibility testing) as one of the 10 most prevalent serotypes in our region, thus warranting further exploration. We initially observed the prevalence of isolates from human blood (374, 39%), stool (116, 12.1%), urine (46, 4.8%), and body fluids (39, 4%). Isolates from food included chicken meat (213, 22.3%) and three isolates from peanuts. The institute received 35 isolates from drag swabs and five from sewage sludge, characterizing them as environmental isolates (40, 4.2%). Moreover, it characterized those from poultry (92, 9.6%) and 34 others (34, 3.6%) from swine, foals, and dogs samples as having an animal origin.

The 957 isolates showing the 10 most frequent serotypes for which antimicrobial susceptibility tests were conducted included Typhimurium, Heidelberg, *S*.I.4,[5],12:i:-, Enteritidis, Mbandaka, Dublin, Infantis, Newport, Typhi, and Saintpaul (Table 1).

**Table 1:** The distribution of a^ntimicrobial susceptibility results by serotype.^

Serotypes	Number of antibiotics															Total
	0	1	2	3	4	5	6	7	8	9	10	11	12	13	14	
Typhimurium	114	18	3	27	6	6	4	9	1							188
Heidelberg	4		2	1	5	5	7	27	34	40	10	14	2	2		153
S.I. 4,5,12:i:-	34	10	4	7	7	11	17	6	8	2	1	1	1		1	110
Enteritidis	12	56	25	3	2		1	1	1							101
Mbandaka	60	4		4	3	2	1							1		75
Dublin	35	9	8	11	4	2	1			1						71
Infantis	47	7	2	3		2		3					2			66
Newport	48	9	5		3								1			66
Typhi	53	10	1													64
Saintpaul	38	19	1	2	1	1			1							63
Total	445	142	51	58	31	29	31	46	45	43	11	15	6	3	1	957

### Antimicrobial resistance profile

Antimicrobial resistance occurred commonly in the investigated *Salmonella* isolates, with 512 of the 957 strains showing it to at least one drug (Supplementary Table S2). A total of 319 isolates (n=957; 33.4%) showed resistance to at least three different classes of drugs and were considered multi-drug resistant (MDR). The Heidelberg and Mbandaka (predominantly found in nonhuman samples) and the Typhimurium serotypes and its monophasic variant (present in both human and nonhuman sources) showed MDR.

Table 1 illustrates the correlation between serotypes and the number of antibiotic resistances in the isolates. It shows that the Heidelberg serotype is correlated with a greater number of antibiotic resistances, whereas *S*.I.4,[5],12:i shows the highest prevalence of resistance to different antibiotics.

*Salmonella* Typhi was the most susceptible serotype, most isolates resisted to only one drug, and one isolate showed resistance to two drugs. *S.* Enteritidis, one of the most frequently isolated serotypes globally and often associated with foodborne outbreaks, shows few resistance markers.

Amikacin, chloramphenicol, cefepime, and trimethoprim/sulfamethoxazole showed the lowest resistance rates (Table 2). Imipenem affected all samples. Considering the source of isolation, environmental isolates, followed by human ones, showed the greatest sensitivity.

**Table 2:** Distribution of antimicrobial susceptibility rates according to isolation source. Shades of green indicate the highest antimicrobial susceptibility rates (color scale at the bottom of the table).

Antibiotic	Overall (n=957)	Source-specific susceptible rate			
		Food (n=216)	Environment (n=40)	Animal (n=126)	Human (n=575)
AMC	84.8%	50.9%	97.5%	77.6%	98.1%
AMI	90.7%	97.7%	60.0%	95.2%	89.2%
AP	72.8%	44.4%	92.5%	67.2%	83.5%
ATM	93.2%	80.1%	100.0%	92.8%	97.9%
CAZ	84.6%	50.0%	97.5%	75.2%	98.8%
CIP	69.2%	52.8%	82.5%	64.8%	75.5%
CO	92.6%	96.8%	100.0%	88.8%	91.5%
CPM	96.7%	94.9%	100.0%	93.6%	97.9%
CRO	84.3%	51.4%	100.0%	74.4%	97.7%
CTX	81.2%	46.8%	90.0%	72.8%	95.5%
ET	43.2%	38.9%	25.0%	44.0%	45.9%
FOX	85.8%	50.5%	97.5%	80.8%	99.3%
GN	83.6%	93.5%	60.0%	82.4%	81.9%
IPM	100%	100.0%	100.0%	100.0%	100.0%
NA	61.5%	38.4%	85.0%	64.8%	67.8%
PE	88%	82.4%	95.0%	90.4%	89.0%
SF	66.4%	43.1%	52.5%	64.0%	76.7%
SFT	95.3%	96.8%	90.0%	88.8%	96.7%
TT	67.7%	44.0%	67.5%	60.0%	78.4%

**Color Scale:**

More susceptible

Less susceptible

AMC = amoxicillin/clavulanic acid; AMI = amikacin; AP = ampicillin; ATM = aztreonam; CAZ = ceftazidime; CIP = ciprofloxacin; CO = chloramphenicol; CPM = cefepime; CRO = ceftriaxone; CTX = cefotaxime; ET = streptomycin; FOX = cefoxitin; GN = gentamicin; IPM = Imipenem; NA = nalidixic acid; PE = pefloxacin; SF = sulfonamide; SFT = trimethoprim/sulfamethoxazole; TT = tetracycline.

The highest rate of resistance occurred for nalidixic acid (357/957; 37.3%), followed by tetracycline (303/957; 31.7%), sulfonamides (281/957; 29.4%) ampicillin (257/957; 26.9%), streptomycin (163/957; 17%), and third-generation cephalosporins, which occurred in 155 isolates (16.2%). All the isolates showed susceptibility to imipenem.

Over the years, a constant rate of antimicrobial susceptibility has been observed for most drugs (Table 3). Notably, the 957 isolates showed a significant reduction in their susceptibility rates to aminoglycoside gentamicin, which raised from about 90% to 20% in the most recent year.

**Table 3:** Distribution of antimicrobial susceptibility rates according to the year of isolation. Shades of green indicate the highest antimicrobial susceptibility rates (color scale at the bottom of the table).

Drug Class	Antibiotic	2016	2017	2018	2019	2020	2021	2022	2023
		(n=105)	(n=117)	(n=206)	(n=129)	(n=137)	(n=107)	(n=64)	(n=92)
	AMI	100.00%	99.10%	100.00%	100.00%	99.30%	100.00%	100.00%	98.90%
Aminoglycosides	ET	47.20%	50.40%	46.60%	45.70%	50.40%	35.80%	28.10%	25.50%
	GN	88.70%	89.70%	92.30%	93.80%	86.30%	96.20%	87.50%	19.10%
β-lactam combination agents	AMC	87.70%	85.50%	58.20%	95.30%	89.20%	95.30%	96.90%	95.70%
Penicillin	AP	68.90%	67.50%	48.60%	86.00%	79.10%	90.60%	76.60%	85.10%
	CAZ	89.60%	84.60%	57.70%	96.10%	84.90%	96.20%	95.30%	93.60%
	CPM	95.30%	98.30%	94.70%	100.00%	95.70%	100.00%	95.30%	98.90%
Cephalosporins	CRO	85.80%	84.60%	59.10%	95.30%	87.80%	98.10%	93.80%	93.60%
	FOX	90.60%	85.50%	58.20%	96.10%	89.20%	96.20%	96.90%	95.70%
	CTX	90.60%	94.00%	70.70%	96.10%	93.50%	97.20%	95.30%	93.60%
Monobactams	ATM	91.50%	97.40%	81.70%	100.00%	96.40%	97.20%	95.30%	95.70%
Carbapenems	IPM	100.00%	100.00%	100.00%	100.00%	100.00%	100.00%	100.00%	100.00%
Quinolones and fluoroquinolones	CIP	90.60%	100.00%	95.70%	99.20%	97.10%	98.10%	100.00%	96.80%
	NA	44.30%	53.80%	38.90%	72.90%	66.90%	86.80%	76.60%	76.60%
Phenicols	CO	85.80%	92.30%	94.70%	93.80%	91.40%	98.10%	90.60%	90.40%
Folate pahtway antagonists	SF	59.40%	69.20%	47.60%	86.80%	77.00%	94.30%	70.30%	31.90%
	SFT	92.50%	97.40%	95.20%	97.70%	95.00%	100.00%	95.30%	88.30%
Tetracyclines	TT	63.20%	66.70%	48.10%	82.20%	71.90%	87.70%	79.70%	59.60%

**Color scale:**

More susceptible

Less susceptible

AMC = amoxicillin/clavulanic acid; AMI = amikacin; AP = ampicillin; ATM = aztreonam; CAZ = ceftazidime; CIP = ciprofloxacin; CO = chloramphenicol; CPM = cefepime; CRO = ceftriaxone; CTX = cefotaxime; ET = streptomycin; FOX = cefoxitin; GN = gentamicin; IPM = Imipenem; NA = nalidixic acid; PE = pefloxacin; SF = sulfonamide; SFT = trimethoprim/sulfamethoxazole; TT = tetracycline.

The disk-approximation test characterized 42 isolates (42/957; 4.4%) showing "ghost zones," suggesting the potential presence of ESBL producers. Regarding the source of ESBL-positive isolates, 17 stemmed from humans, namely: *S*. Typhimurium (5), its monophasic variant (3), Saintpaul (4), and Dublin (2). Conversely, of the nonhuman isolates, the prevalent serotypes tested positive for ESBL included Heidelberg (12), followed by Infantis (5), monophasic *S*. Typhimurium (2), and Mbandaka (2).

Disk diffusion testing identified 123 cefoxitin-resistant *S*. Heidelberg isolates (123/957; 12.9%). Despite the exclusion of the Minnesota serotype due to its absence among the top 10 most frequent serotypes, 48 cefoxitin-resistant isolates out of 57 (48/57; 84.2%) underwent antibiotic susceptibility testing.

In total, 29 isolates showed resistance to ciprofloxacin (3%) and 266 (27.8%), reduced susceptibility to it. However, 115 isolates (12%) resisted the antibiotic pefloxacin.

Regarding *Salmonella* serovars, MDR isolates (resistant to three or more classes of antibiotics), mainly occurred for *Salmonella* Heidelberg (147/319), *S*. Typhimurium (53/319) and its monophasic variant (62/319). *Salmonella* Heidelberg showed its highest resistance levels against nalidixic acid (96.7%), sulfonamide (94.1%), tetracycline (92.8%), ampicillin (90.2%), and cefoxitin (80.4%). Out of the 147 MDR isolates of *S*. Heidelberg, 115 isolates stemmed from food; 31, from animals; and only one, from humans.

Most *Salmonella* Typhimurium strains (n=188) showed resistance to streptomycin (52; 27.7 %) tetracycline (46/188; 24.5%), followed by sulfonamide (46/188; 24.5%) and ampicillin (24/188; 12.8%). Its monophasic variant strains (n=110) showed resistance to tetracycline (62; 56.4%), nalidixic acid (56/110; 51%), ampicillin (55/110; 50%), and sulfonamide (44/110; 40%). Considering the MDR strains, we found 53 isolates of MDR *S*. Typhimurium, with 37 originating from humans; 11, from the environment; three, from food; and two, from animals. As for its monophasic variant MDR (n=62), 53 resistant isolates originated from humans; 6, from animal sources; 2, from the environment; and 1, from food.

*Salmonella* Enteritidis (n=101) showed the highest resistance against nalidixic acid (83/101; 82.2%) and pefloxacin (24/101; 23.8%). Of the six MDR isolates, five originated from human sources and one from an animal source.

*S*. Dublin only stemmed from human isolates. Its 71 isolates included 17 (24%) MDR ones. They showed resistance to tetracycline (23/71; 32.4%), ampicillin (17/71; 24%), and streptomycin (7/71; 9.9%). *Salmonella* Typhi, a host-specific serotype, showed no antimicrobial resistance markers.

Of the 144 *S.* Heidelberg isolates, 29 (20.1%) showed resistance profiles to nalidixic acid, amoxicillin/clavulanic acid, ampicillin, ceftazidime, ceftriaxone, cefoxitin, cefotaxime, sulfonamide, and tetracycline; 28, (19.4%) to all of these, except ceftriaxone; 21, (14.6%) to nalidixic acid, amoxicillin/clavulanic acid, ampicillin, cefoxitin, CTC, sulfonamide, and tetracycline. In total, 21 *S*. Typhimurium isolates showed resistance profiles to streptomycin, sulfonamide, and tetracycline and five, to nalidixic acid, ampicillin, chloramphenicol, streptomycin, pefloxacin, sulfonamide, and tetracycline. In total, five monophasic isolates showed resistance to nalidixic acid, ampicillin, chloramphenicol, streptomycin, gentamicin, pefloxacin, sulfonamide, and tetracycline, whereas six, to nalidixic acid, ampicillin, gentamicin, sulfonamide, and tetracycline.

## DISCUSSION

This study identified, amidst a large collection of *Salmonella* serotypes, trends of higher resistance in isolates from animal and food sources. The increase of antibiotic resistance significantly challenges global public health and stress the urgent need to understand the true extent of this resistance, particularly in regions with limited surveillance and sparse data^11,12^. Antimicrobial resistance in *Salmonella* varies by serotype and by source and geographical location^3-4^. It is crucial to emphasize that the focus should extend beyond humans and include animal-origin foods as *Salmonella* is a foodborne pathogen^1^.

Of the top 10 *Salmonella* serotypes involved in human infections, *S*. Typhimurium, *S*.I. 4,5,12:i:-, and *S*. Enteritidis emerged as the most prevalent ones, consistently detected throughout the study period and in line with previous findings worldwide. Conversely, in nonhuman sources, *S*. Heidelberg, *S*. Mbandaka, and *S*. Typhimurium have predominated as the three most common serotypes as they have been found worldwide^13-15^.

This study observed a wide range of diverse resistance patterns. Investigation into the susceptibility of *Salmonella* serotypes to antibiotics indicated that most strains showed resistance to at least one drug, with its highest levels of resistance referring to nalidixic acid, streptomycin, sulfonamides, and tetracycline. These high levels of resistance agree with previous reports^16-18^. However, recent years have seen an increase in susceptibility to nalidixic acid (Table 3). This shift maybe attributed to preventive strategies to combat antimicrobial resistance, particularly in food and animal samples. Several countries have restricted or banned the use of antimicrobials in food animals as growth promoters. Additionally, improvements in hygiene and feed management have been reported to mitigate the negative impacts of such bans on animal health and productivity. Reducing the use of unnecessary antimicrobial agents is crucial to prevent the emergence and spread of drug-resistant bacteria^6,12,18^.

A study with poultry in southern Brazil from 2014 to 2017 found resistance to nalidixic acid, ampicillin, cefotaxime, ceftazidime, ciprofloxacin, and tetracycline. A significant increase in resistance to these antibiotics has been observed more recently. Multidrug resistance occurred in 50.7% (74/146) of the isolates from 2014, increasing to 77.3% (126/163) in 2017^19^. Another study, conducted in the Federal District, Brazil, with chicken meat showed higher resistance to amoxicillin/clavulanic acid (83.3%), followed by sulfonamide (64.1%) and tetracycline (46.2%); 53.8% of the isolates were MDR^20^. A meta-analysis in Brazil assessed the antimicrobial resistance of nontyphoidal *Salmonella* that had been isolated from poultry from 1995 to 2014. The highest levels of resistance referred to sulfonamides (44.3%), nalidixic acid (42.5%), and tetracycline (35.5%)^16^. In Brazil, a notable 80.9% of *Salmonella* isolates from different stages of the pork production chain showed multidrug resistance (to ≥3 antibiotic classes). The highest resistance rates occurred for streptomycin (90.5%), tetracycline (88.1%), ampicillin (81.0%), chloramphenicol (71.4%), and ciprofloxacin (50.0%)^21^.

Brazil has regulations that prohibit the use of antibiotics such as chloramphenicol, colistin, erythromycin, tetracyclines, fluoroquinolones, beta-lactams, and sulfonamides as additives or growth promoters, restricting their use to therapeutic purposes^22,23^. However, these medications still apply selective pressure on microorganisms.

The traditional primary antimicrobial choices to treat *Salmonella* infections included ampicillin, trimethoprim-sulfamethoxazole, and chloramphenicol^2,24^. However, widespread resistance has rendered these options less effective. Currently, recommendations suggest the use of fluoroquinolones, azithromycin, and third-generation cephalosporins as alternatives^25,26^. In 2024, WHO listed critically important antimicrobials, such as third and fourth-generation cephalosporins, fluoroquinolones, and macrolides, alongside highly important antimicrobials like chloramphenicol and sulfonamides^27^.

Fluoroquinolones, in turn, serve as the gold standard for treating invasive salmonellosis in human medicine, whereas veterinary medicine extensively uses ampicillin and tetracycline as primary treatments^28-30^. However, the literature describes a great number of isolates with decreased susceptibilities to fluoroquinolones and fluoroquinolone-resistant *Salmonella* strains^28-30^. This study detected a high frequency of isolates with resistance to nalidixic acid and reduced susceptibility to ciprofloxacin. Treatments with fluoroquinolones have failed in patients infected with *Salmonella* spp., attributed to single point mutations in the quinolone-resistance determining region and plasmid-mediated resistance mechanisms. This issue is considered a serious public health concern worldwide^30^. Here, we can observe that pefloxacin shows better results in screening isolates, better separating isolates that are susceptible to ciprofloxacin from those that resist it. A study on chicken carcass samples from Rio de Janeiro, Brazil, found that all strains showed resistance to at least one antimicrobial in the quinolone class. Specifically, 100% of the isolates resisted nalidixic acid and enrofloxacin, whereas 63.64%, ciprofloxacin^31^. Another study on poultry meat in Brazil assessed samples from 2014 to 2017 and showed high resistance rates to nalidixic acid and ciprofloxacin among *Salmonella* isolates from poultry^19^. A study in our laboratory over a five-year period in Sao Paulo State, Brazil, quinolone susceptibility testing of *Salmonella* strains showed resistance to NAL and reduced susceptibility to ciprofloxacin. Ciprofloxacin-resistant strains occurred in the Enteritidis, Typhimurium, *S*. I. 4,5,12:i:-, and Heidelberg serotypes, which are commonly associated with human infections and poultry isolates in Brazil^32^. A study in China observed the prevalence of *qnr*-positive *Salmonella* strains in chickens and their carriage of multiple resistance traits. The emergence and increasing prevalence of the FQ-resistant gene *qnr* in *Salmonella* have been widely isolated from chickens^30-32^. A study, carried out in Poland from 2018 to 2019, detected fluoroquinolone resistance most frequently in several serotypes such as Hadar, Virchow, Newport, Infantis, Enteritidis, monophasic 1,4,[5],12:i:-, and Typhimurium, which commonly occur in humans. Results indicated a high level of FQ resistance (37.6%) in the tested isolates^33^. In the European Union, significant resistance to fluoroquinolones (ciprofloxacin) has been detected in isolates from broilers (55.5%), fattening turkeys (57.9%), and laying hens (24.7%) in 2022. For human *Salmonella* isolates reported in the same year, the overall ciprofloxacin resistance rate totaled 18.7%, with the lowest resistance in monophasic *S*. Typhimurium (9.6%) and the highest in *S*. Infantis (40.1%) and *S*. Kentucky (72.7%). Analysis of resistance trends from 2013 to 2022 showed significant increases in nine countries and decreases in three, with the most pronounced rises in *S*. Enteritidis, *S*. Typhimurium, its monophasic variant, and *S*. Infantis^18^. These results cause concern as *S*. Enteritidis and *S*. Typhimurium are associated with foodborne outbreaks and human infections worldwide^15^.

Third-generation cephalosporins serve to treat human infections when fluoroquinolones are not recommended (such as during childhood infections). This study found that 16.2% of its samples resisted cephalosporins, with 4.4% showing an ESBL phenotype. Additionally, the 12.9% of *S*. Heidelberg isolates resisted cefoxitin, suggesting a possible ampC-type ESBL phenotype, such as in studies in Brazil and worldwide^34,35^. Notably, the resistance profile NAL-AMP-CTX-CAZ-CIP-TET prevailed in poultry isolates from southern Brazil, accounting for 26.0% (38/146) in 2014 and 63.2% (103/163) in 2017. These results are associated with the *Salmonella* Heidelberg and Minnesota serotypes in this region and with poultry isolates^19^. In Another study, carried out in Brazil from 2004 to 2011, found isolates from poultry carrying specific genetic variants of *bla*_CTX-M_ across three regions, indicating possible clonal dissemination^36^. In a recent study, conducted on chicken carcasses in Rio de Janeiro, Brazil, with samples collected from 2016 to 2022, phenotypic tests for ESBL production showed that 36.36% (4/11) of the strains were positive^31^. A study analyzing antimicrobial resistance profiles using 191,306 publicly available *Salmonella* whole-genome sequencing (WGS) data identified the most common β-lactam resistance gene profiles as *bla*_TEM-1B_ (6.78%), *bla*_CMY-2_ (2.82%), and *bla*_CTX-M-65_ (1.68%)^37^. The *bla*_TEM-1B_ profile was dominantly harbored in *Salmonella* isolates worldwide. A study on *Salmonella* isolates from sporadic diarrhea cases in China (2014-2021) showed a 19.3% resistance rate to third-generation cephalosporins, with specific resistance rates of 10.4% to ceftazidime and 19.1% to cefotaxime, indicating ESBL production. However, the absence of an increasing trend in resistance suggest that these antimicrobials remain effective for most *Salmonella* infections^38^. Our data indicate moderate resistance proportions for cephalosporins. Importantly, we found no carbapenem-resistant isolate. Despite a decline in isolates showing the ESBL phenotype over time, the concerning presence of these resistances in both human and nonhuman isolates to these antibiotic classes throughout the studied period underscores the imperative for ongoing efforts in antibiotic surveillance.

Decreasing trends in resistance occurred more commonly for ampicillin and chloramphenicol antibiotics in *Salmonella* spp. Despite this decline, resistance to these antibiotics remains high in bacteria isolated from humans and animals. Increasing trends of resistance commonly occurred for streptomycin, gentamicin, sulfonamides, and tetracycline. This pattern of antibiotic resistance has been observed in *Salmonella* isolated from the food chain^39^. We hypothesize that the substantial use of antimicrobials in food-producing animals plays a crucial role in generating antimicrobial residues and contributing to the global burden of antimicrobial resistance, potentially maintaining or even increasing resistance levels for certain drugs. The rise of gentamicin resistance, notably accentuated in recent times, is a significant observation. This trend was particularly pronounced in Canada following the classification of gentamicin as a category II antimicrobial by the Canadian Veterinary Drugs Directorate, ultimately leading to its prohibition by the end of 2018^5^. The Canadian Integrated Program for Antimicrobial Resistance Surveillance documented a continuous rise in the prevalence of gentamicin resistance among *Salmonella* *enterica* isolates that had been sourced from human infections and broiler chicken populations^5^. A hypothesis suggests that the increased use of the lincomycin-spectinomycin combination in poultry farming might inadvertently contribute to the development of gentamicin resistance^5^.

In Europe, the MDR analysis of animal isolates included the following antimicrobials: amikacin/gentamicin (for pigs and calves) or gentamicin only (for poultry populations), ampicillin, cefotaxime/ceftazidime, chloramphenicol, ciprofloxacin/nalidixic acid, meropenem, sulfamethoxazole, tetracycline/tigecycline, and trimethoprim^18^. Concerning MDR strains, our findings evince that 32.8% of *Salmonella* strains show resistance to over three antibiotic classes. This study highlights that MDR prevalence is most pronounced in *S*. Heidelberg, *S*. Typhimurium, including its monophasic variant, and *S.* Dublin, with elevated resistance levels to nalidixic acid, sulfonamides, streptomycin, and tetracycline, consistent with reports from other countries^16,18-39^. While resistance rates differ among serotypes and antibiotics, *S. enterica* serotype Enteritidis, one of the most prevalent serotype, is relatively more susceptible to antimicrobial agents compared to others. Conversely, *S. enterica* serotype Typhimurium shows a much higher resistance rate, being another globally prevalent serotype^2,18^.

## CONCLUSION

*Salmonella* constitutes one of the leading causes of human death worldwide due to diarrheal diseases worldwide. Understanding the epidemiological status of *Salmonella* is thus crucial to control this pathogen. Monitoring programs, prudent use guidelines, and educational campaigns provide approaches to minimize the further development of antimicrobial resistance and to control the spread of antibiotic resistant bacteria.

## Data Availability

https://doi.org/10.48331/scielodata.GD4O4J
